# Digital Epidemiological Approaches in HIV Research: a Scoping Methodological Review

**DOI:** 10.1007/s11904-023-00673-x

**Published:** 2023-11-02

**Authors:** Lindsay E. Young, Yuanfeixue Nan, Eugene Jang, Robin Stevens

**Affiliations:** Annenberg School for Communication and Journalism, University of Southern California, 3502 Watt Way, Los Angeles, CA 90089 USA

**Keywords:** HIV, Digital epidemiology, Infodemiology, Infoveillance, Digital public health surveillance, Digital data

## Abstract

**Purpose of Review:**

The purpose of this scoping review was to summarize literature regarding the use of user-generated digital data collected for non-epidemiological purposes in human immunodeficiency virus (HIV) research.

**Recent Findings:**

Thirty-nine papers were included in the final review. Four types of digital data were used: social media data, web search queries, mobile phone data, and data from global positioning system (GPS) devices. With these data, four HIV epidemiological objectives were pursued, including disease surveillance, behavioral surveillance, assessment of public attention to HIV, and characterization of risk contexts. Approximately one-third used machine learning for classification, prediction, or topic modeling. Less than a quarter discussed the ethics of using user-generated data for epidemiological purposes.

**Summary:**

User-generated digital data can be used to monitor, predict, and contextualize HIV risk and can help disrupt trajectories of risk closer to onset. However, more attention needs to be paid to digital ethics and the direction of the field in a post-Application Programming Interface (API) world.

**Supplementary Information:**

The online version contains supplementary material available at 10.1007/s11904-023-00673-x.

## Introduction

More than 40 years after the first official reporting on what became known as the AIDS (acquired immunodeficiency syndrome) epidemic [1], human immunodeficiency virus (HIV) remains a worldwide public health concern, with approximately 1.5 million new cases reported globally in 2021 alone [2]. Biomedical advances in antiretroviral therapy (ART) for HIV management and pre-exposure prophylaxis (PrEP) for HIV prevention have made considerable inroads in the fight against HIV. However, these tools have been underutilized in some of the most HIV-susceptible and socially marginalized populations [3–5], for example Black and Latinx men who have sex with men (MSM), transgender women, and people who inject drugs. To address these disparities, high-impact initiatives like the US Ending the HIV Epidemic initiative and UNAIDS’s 95–95-95 program have set ambitious targets to expand access to ART for persons with HIV and to PrEP for those at high risk of HIV acquisition [6, 7].

Whether these initiatives succeed depends on timely and accurate HIV surveillance. Yet, as others have argued, the problem with existing surveillance systems is that they are resource-intensive and inherently backward-looking [8•, 9]. For example, although documentation of new HIV diagnoses in the USA is near complete, these “new” diagnoses often reflect infections that occurred months prior. Additionally, behavioral surveillance surveys rely on self-reports of past behaviors. Consequently, these systems are poorly suited for the deployment of timely interventions capable of disrupting trajectories of HIV risk closer to their onset. Moreover, the surveys and interviews on which traditional surveillance strategies rely are also prone to reporting biases, including socially desirable responses, which is particularly problematic given that HIV prevalence is higher among those who engage in stigmatized sex and drug use behaviors [9]. Finally, with studies suggesting limited access to health care services for many high-risk subgroups, data predicated on an individual’s utilization of healthcare services may not represent those at greatest risk for contracting or unknowingly transmitting HIV nor those who are most vulnerable to falling out of care once diagnosed.

The global explosion of internet, mobile phone, and social media usage has ushered in new opportunities for disease surveillance, prevention, and treatment that have potential for overcoming some of the limitations outlined above. As a consequence of the rapid expansion of digital communication technologies, much of what individuals do and say about a wide variety of topics, including aspects of personal and communal health, is stored and shared electronically, often in forms accessible to third parties and thus amenable to analysis [8•]. The extraction of epidemiologically relevant information from these data and the timely incorporation of such data into disease surveillance systems is the objective of *digital epidemiology*. Like traditional epidemiology, digital epidemiology aims to understand the who, what, and when of disease in a defined population, as well as its determinants, but does so using user-generated digital data that was not generated with the primary purpose of doing epidemiology [10]. Using geotagged social media posts about substance use to predict overdose hospitalizations, mobile phone call data to trace spatial mobility patterns during a pandemic, and internet search queries to locate disease outbreaks exemplify the potential of this approach.

The promise of digital epidemiology for HIV research has been extolled in several commentaries over the past decade [9, 11–13]. However, to date, we lack a comprehensive review of the empirical work adopting these techniques. In this paper, we perform a scoping review to evaluate the nature and range of digital epidemiological approaches applied in HIV surveillance, prevention, and treatment research. Given that more than two decades have passed since the publications of some of the earliest digital epidemiology papers [14, 15] and that digital technologies and methods have advanced considerably during that time, now seems like an opportune moment to engage in such a review.

## Methods

To guide this review, we used the scoping review methodology outlined by Arksey and O’Malley [16], which involved five stages: (1) identification of a research question; (2) identification of relevant articles; (3) selection of articles; (4) extraction and charting of data; and (5) synthesizing, summarizing, and reporting results.

### Search Strategy

Our search included studies published as of July 2023. We did not set a lower bound limit as the field of digital epidemiology is relatively young and we wanted to determine when HIV studies using these methods first emerged. On July 18, 2023, we searched PubMed, Scopus, and Web of Science databases. We also used Google Scholar to locate additional relevant studies identified from reference lists and an expert in the field. Although we privilege the term “digital epidemiology” to describe our methodological field of interest, we consider it an umbrella term for other analogous terms, including “digital public health surveillance” and Eysenbach’s [17•] preferred terms, “infodemiology” and “infoveillance.” As such, we searched each database using the two keyword Booleans shown in Table 1.

**Table 1:** Search keyword Booleans

	Search fields	Keyword Boolean
Search 1	Title/Abstract	[(“human immunodeficiency virus” OR “HIV”) AND (“digital epidemiolog*” OR “digital public health” OR “infodemiolog*” OR “infoveillance”)]
Search 2	Title/Abstract	[(“human immunodeficiency virus” OR “HIV”) AND (“epidemiolog*” OR “disease surveillance”) AND (“smartphone data” OR “mobile phone data” OR “spatial mobility” OR “geolocation data” OR “social networking data” OR “social media data”)]

## Inclusion and Exclusion Criteria

Studies were included if (1) their primary focus was on the epidemiology of HIV, which included studies pertaining to HIV surveillance, risk monitoring, and the identification of determinants, mechanisms, and social contexts associated with HIV, and (2) the analysis featured user-generated digital data. We excluded studies not published in English, dissertations and theses, reviews and protocols, and any studies where digital epidemiology for HIV was not its primary focus.

## Screening and Article Classification

After removing duplicates, the titles and abstracts of the remaining articles were evaluated for relevance by reviewers YN and EJ. Reviewer RS settled any uncertainties. From the studies deemed relevant, data were independently extracted by reviewers LY, YN, and EJ. To guide data extraction, we examined two scoping reviews of digital epidemiology broadly [18, 19] to identify study features of interest. Table 2 describes the pieces of information we extracted. Specific categories for each variable were first characterized inductively by reviewers LY, YN, and EJ and then regrouped post hoc into logical higher-level categories by reviewer LY. Table 2 describes these variables in more detail.

**Table 2:** Definitions of variables

Variable	Definition	Variable categories
Year of publication	Year in which the article was published	From 2013 to 2023
Geographic region of study	Region included in the study	Africa Asia Europe North America South America Global Unspecified
Priority populations	HIV priority populations addressed by the study	Men who have sex with men Transgender people People who inject drugs Youth/young adults (aged 13–24)
Sources of digital data	Types of digital data studied	Web search query Social media Mobile phone data Other
Epidemiological purpose	The epidemiological need met through the analysis of digital data	Disease surveillance Behavioral surveillance Assess public attention Characterize risk contexts
External data	Study uses ground truth data (not user-generated)	Yes No
Outcome measures	The nature of the outcome measure or primary feature being described (for descriptive studies)	HIV incidence, prevalence, infection HIV prevention/care engagement Sexual risk behaviors Feature of the search query Feature of the social media post Feature of network contexts Feasibility/acceptability
Analytic purpose	The objective of performing the analysis	Describe Explore Explain Predict
Analytic methods	Statistical and other analytic techniques used in the study	Descriptive statistics Tests of difference Correlation analyses Regression analyses Disease modeling Spatial modeling Network modeling Topic modeling Machine learning
Consideration of digital ethics	Authors discussed the ethics of using user-generated digital data	Yes No

## Results

**Fig. 1:**
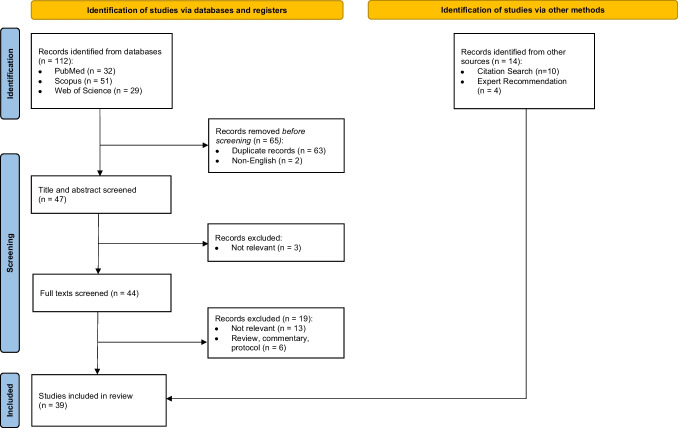
PRISMA flowchart

Figure 1 depicts the results of the screening and article selection process as a PRISMA flowchart [20]. As a result of our keyword search, we identified 112 articles, 65 of which were duplicates or non-English and were therefore removed. In total, 44 articles underwent full text screening, after which 19 were excluded for being not relevant. An additional 14 articles were identified from references or expert recommendation, yielding a total of 39 articles included in this review. The full list of relevant studies is provided in Supplemental Table 1.

### Study Sample

The articles included in the sample were published between 2013 and 2023. Approximately half of the articles (*n* = 20) had been published in the last 4 years (2020 to 2023). The geographic distribution of the included studies was biased toward North America (*n* = 19), nearly all of which were conducted in the USA. The remaining 20 articles focused on the HIV epidemic in Sub-Saharan Africa (*n* = 6), South and East Asia (*n* = 5), Russia (*n* = 2), and Brazil (*n* = 1). Six more articles were agnostic to geography. Finally, 12 studies paid unique attention to high-priority subpopulations, including MSM (*n* = 10), transgender women (*n* = 2), people who inject drugs (*n* = 2), and youth and young adults (*n* = 1).

### Study Characteristics

Study characteristics are summarized in Table 3, which also includes reference numbers for the studies that possess each characteristic.

**Table 3:** Characteristics of the studies reviewed (*n* = 39)

Category	Number (*n*)	Percent (%)	References
Sources of digital data			
Social media	21	53.8	[21, 22•, 23–27, 38, 39, 40•, 41, 42, 44•, 49•, 50, 52•, 54–58]
Web search query	10	25.6	[34, 35, 45, 47•, 48, 51, 59–62]
Mobile phone data	6	15.4	[28•, 29–33]
Other GPS technology	2	5.1	[36, 37]
Epidemiological purpose			
Disease surveillance	15	38.5	[24, 28•, 29, 30, 34, 38, 44•, 47•, 48, 49•, 51, 55, 58–60]
Behavioral surveillance	8	20.5	[22•, 31–33, 40•, 42, 52•, 57]
Assess public attention	12	30.8	[23, 25–27, 35, 39, 41, 45, 50, 54, 61, 62]
Characterize risk contexts	4	10.2	[21, 36, 37, 56]
External Data			
Yes	28	71.8	[21, 22•, 24, 28•, 29–31, 33–36, 38, 39, 40•, 44•, 45, 47•, 48, 49•, 51, 54, 55, 57–62]
Outcome measures^a^ (*n* = 30)			
HIV incidence, prevalence, infection	16	53.3	[24, 28•, 29, 34, 38, 44•, 45, 47•, 48, 49•, 51, 55, 56, 58–60]
HIV prevention/care engagement	3	10.0	[22•, 30, 39]
Sexual risk behaviors	2	6.7	[22•, 33]
Feature of the search query	4	13.3	[35, 60–62]
Feature of the social media post	5	16.7	[24, 40•, 42, 52•, 57]
Feature of network contexts	3	10.0	[21, 31, 57]
Feasibility/acceptability	2	6.7	[30, 36]
Analytic purpose			
Describe	7	17.9	[23, 26, 27, 32, 41, 50, 54]
Explore	15	38.6	[22•, 24, 25, 30, 36, 37, 39, 42, 52•, 56–58, 60–62]
Explain	7	17.9	[21, 29, 31, 35, 45, 49•, 55]
Predict	10	25.6	[28•, 33, 34, 38, 40•, 44•, 47•, 48, 51, 59]
Analytic methods^a^			
Descriptive statistics	8	20.5	[23, 25, 30, 36, 37, 39, 50, 54]
Tests of difference	4	10.3	[24, 25, 54, 61]
Correlation analyses	9	23.1	[25, 34, 39, 48, 49•, 59–62]
Regression analyses	20	51.3	[22•, 24, 28•, 33–36, 38, 40•, 42, 44•, 45,47•, 48, 49•, 51, 55, 58–60]
Network modeling	7	17.9	[21, 28•, 31, 32, 52•, 56, 57
Topic modeling	4	10.3	[26, 27, 40•, 41]
Disease modeling	2	5.1	[29, 56]
Machine learning	12	30.8	[26, 27, 28•, 38, 40•, 41, 42, 44•, 47•, 50, 51, 52•]
Digital ethics			
Yes	8	20.5	[21, 22•, 23, 24, 33, 36, 52•, 57]

^a^Multiple responses

#### Sources of Digital Data

Four types of digital data were leveraged in the studies we reviewed, including social media data (53.8%), web search queries via Google, Yandex, and Baidu (25.6%), mobile phone data (15.4%), and data from global positioning system (GPS) devices other than mobile phones (5.1%). Of the 21 studies that analyzed social media data, 17 examined publicly available data on Twitter. Exceptions to this were two studies by Young et al. [21,22•] that drew exclusively on Facebook data collected with consent from a cohort of sexual minority men, and one study each that leveraged data from Reddit [23] and Baidu Tieba [24], a Chinese social media platform. Three more studies incorporated data from multiple platforms [25–27] (i.e., various combinations of data from Twitter, Reddit, Instagram, YouTube, and Tumblr). Irrespective of the platform, 20 of the 21 social media studies analyzed post content. Far fewer examined alternative sources of information, such as social relationships among users, hashtags, or URLs. Among the six studies that leveraged mobile phone data, all but one used that data to map human spatial mobility [28•, 29–32]. The exception was a study by Kapur et al. [33] that used digitally extracted cell phone contact lists of a sample of high-risk men in India and information about those contacts’ sex behaviors to infer respondents’ sexual risk behaviors.

#### Epidemiological Purpose

We identified four epidemiological needs met by analyses of digital data: disease surveillance (38.5%), behavioral surveillance (20.5%), surveillance of public attention and sentiment (30.8%), and characterization of social contexts (10.2%). Studies designed to meet disease surveillance needs are those that drew on digital data sources to locate and describe HIV burden, to monitor trends in that burden, or to identify HIV outbreaks. For example, Mavragani et al. [34] used search traffic data from Google Trends to forecast AIDS prevalence in US states. Studies with a behavioral surveillance focus drew on digital data with the intention of monitoring behavioral risk factors associated with HIV, such as sexual and drug use behaviors. As an example, Young et al. [22•] demonstrated person-centered associations between Facebook communication themes and patterns of friendship connections and self-reported sex drug use and condomless sex behavior. Other studies used digital data to characterize public attention to HIV with the future goal of being able to utilize those insights to inform health messaging and intervention. For example, Chiu et al. [35] examined the relationship between HIV/AIDS related news coverage and HIV/AIDS related web search queries in Hong Kong, demonstrating windows of opportunity for health agencies to engage in timely health messaging. Finally, several studies used digital data to characterize social contexts associated with HIV prevention and risk behaviors, for example Duncan et al.’s [36, 37] use of geolocation data to map the activity spaces of sexual minority men in the Deep South and New York City with the goal of informing intervention opportunities in these high prevalence regions.

#### External Data

Most articles (71.8%) used ground truth data (i.e., information that is known to be real or true, provided by direct observation and measurement) in their analyses, for example HIV surveillance reports published by the CDC, data aggregators like AIDSVu, clinical data (e.g., lab results), and behavioral surveys. For example, Stevens et al. [38] used the CDC AtlasPlus data platform to obtain estimates of new HIV infections at the county-level and U.S. Census and American Community Survey data to characterize the relationship HIV incidence and risk-specific Twitter activity while adjusting for county-level sociodemographic traits.

#### Outcome Measures

Thirty of the 39 studies included outcome measures. Three types of outcomes were identified. The first type was an observed measure of health status or health behavior. Most common in this category of outcomes were aggregate measures of HIV incidence, diagnosis, or prevalence (53.3%). However, three studies examined outcomes related to HIV prevention and care engagement, namely HIV testing [30, 39] and a status neutral measure of linkage to care [22•], and two more studies examined self-reported sex behaviors [22•, 33]. The second type of outcome was a feature of the digital data itself, including features of social media posts or web search queries (30.0%) or the structure of digitally instantiated networks (10.0%). For example, Cuomo et al. [40•] built a classifier using routinely collected demographic data to accurately predict the occurrence of risk-related tweets at the census block level during the 2015 HIV outbreak in Indiana. And, a study by Young et al. [21] modeled digital connections among sexual minority men in relation to their HIV prevention and risk behaviors to identify viable clusters for intervention. Finally, two pilot studies [30, 36] also assessed feasibility and acceptability outcomes related to a novel digital measurement procedure. For example, Nsabimana et al. [30] assessed the feasibility of using a mobile phone app to track HIV test results in real time and with geospatial context in urban and rural locations in Rwanda.

#### Analytic Purpose

Our analysis revealed four types of analytic purposes: describe (17.9%), explore (38.6%), explain (17.9%), and predict (25.6%). Descriptive studies aim to characterize a population, situation, or phenomenon with empirical data, often by reporting distributions of one or more variables. Here, descriptive studies often characterized HIV-related digital content, most notably search queries or social media posts. For example, Cai et al. [41] used natural language processing (NLP) techniques to characterize geotagged user-generated Twitter messages related to opioid abuse, injection drug use, and HIV status during the 2015 HIV outbreak in Indiana and reported frequencies of tweet themes and the geographic distribution of opioid, heroin, and HIV tweets across counties in Indiana.

Exploratory studies investigate new or under studied phenomena and often lay groundwork for more hypothesis-driven research in the future. Two types of exploratory studies emerged from our analysis. The first type advanced the prototypical descriptive study that simply characterized HIV-related social media or web search query content by correlating the prevalence of that content with metrics of HIV prevalence, incidence, or infection in a specific region. For example, Dong et al. [24] examined HIV-related posts on the Chinese Baidu Tieba platform, created word clouds and codified themes to understand the needs of people living with HIV/AIDS, and assessed relationships between word cloud geolocations and the prevalence of MSM living with HIV/AIDS in local provinces. The second type of exploratory study assessed the feasibility of a particular digital epidemiological approach. As an example, drawing on a large annotated set of Tweets about HIV risk, Young et al. [42] tested the feasibility of using machine learning classifiers to learn patterns of speech and language associated with HIV risk behaviors with the future goal of finding ways to incorporate these models into real-world HIV surveillance systems.

Explanatory studies aim to explain why or how a previously studied phenomenon takes place by establishing underlying causes of the phenomenon or the systematic relationships among variables [43]. Two types of explanatory studies emerged. The first type was theory-driven, in that the selection of key variables in the model was driven by a theoretical framework. For example, Ireland et al. [44•] drew on theory from personality psychology to investigate the relationship between the use of action language in Twitter posts aggregated at the US county level and HIV prevalence, and Park [45] applied a socio-technical and digital equity framework to examine regional differences in the relationship between HIV-related information seeking by US Metropolitan area and HIV prevalence. The second type of explanatory study in this review was less theory-driven but nonetheless focused on mechanisms. For example, Isdory et al. [29] drew on mobile phone data to determine the effect of human spatial mobility between regions in Kenya on HIV transmission.

Finally, predictive studies draw on historical or current data to predict a future phenomenon. Key features of predictive studies are predictors that precede the outcome in time and evaluations of how well the predictive model performs in out-of-sample predictions [46]. Moreover, in the era of “Big Data,” predictive models increasingly feature large numbers of features (or predictors) as the objective is to increase the predictive accuracy of the model as opposed to testing specific hypotheses. As an example of this approach, Brdar et al. [28•] extracted over 200 features from spatial mobility and connectivity traces from mobile phone service data and used them to train and test a machine learning regression model to predict HIV prevalence rates in Ivory Coast. And Young et al. [47•] tested and trained a machine learning regression model to predict HIV diagnoses at the US state level with search volume data on HIV-related Google search keywords.

#### Analytic Methods

A variety of analytic techniques were used, ranging from basic descriptive statistics to advanced machine learning. Overall, a majority of studies (51.3%) used regression analyses, of which linear, negative binomial, and multi-level (mixed effects) models were most common. To account for the role of geography, two studies [48, 49•] used geometrically weighted regression, a modification of traditional regression that can account for geographic variability in the relationship between HIV health outcomes and digital content.

Additionally, several studies drew on specialized modeling approaches, such as network modeling (15.4%), topic modeling (7.7%), and disease modeling (5.1%). In total, seven studies leveraged social network methods to construct and analyze network contexts of HIV risk and transmission, for example, as Valdano et al. [31] did in their study of risk flow networks underlying the HIV epidemic in Namibia. Four studies used topic modeling to detect patterns in social media posts. As an example, Xu et al. [27] used the Biterm Topic Model to detect and characterize barriers to pre-exposure prophylaxis (PrEP) therapy from a large corpus of social media posts across multiple platforms. And, two studies incorporated digital data into compartmental models, the workhorses of infectious disease modeling. For example, Isdory et al. [29] built an SIR metapopulation model parameterized using census data, HIV data, and mobile phone data adopted to track human mobility.

Finally, given the volume and complexity of data that many studies featured, 30.8% of studies used modalities of machine learning, most notably in the form of machine learning classifiers, prediction models, and unsupervised topic models. For example, Adrover et al. [50] drew on boosted decision tree, support vector, and artificial neural network classifiers to identify Twitter posts that conveyed adverse effects of HIV drug treatment. And Zhang et al. [51] trained and tested nowcast and forecast models to estimate the number of new HIV diagnoses in China with web search query data and historical records at the national and provincial levels.

#### Consideration of Digital Ethics

Overall, eight (20.5%) studies included an explicit discussion of the ethical considerations authors made when using user-generated digital data in their study. The most robust of those discussions came from Weibel et al. [52•]. Following their presentation of research, which aimed to identify HIV at-risk populations by exploiting Twitter post, geolocation, and social network data, Weibel et al. walked the reader through their own reflection on the ethics of using social media data for HIV risk research. In this reflection, they outlined the considerations pertaining to data collection and analysis, making model-driven inferences, and designing data-driven interventions.

## Discussion

The purpose of this scoping review was to synthesize and evaluate the state-of-the-field of digital epidemiological approaches in HIV research and to identify areas for further development. At a high level, our search yielded 39 studies published in the last decade that spanned five continents and 11 countries with contributing authors from diverse disciplines including but not limited to health sciences, psychology, computer science and engineering, and communication studies. Multiple types of user-generated data were featured, including web search queries, social media data, mobile phone data, and data from GPS devices. A range of population-level characteristics were gleaned from these data—e.g., about sex and drug use behavior, information-seeking and sentiment about HIV prevention and treatment, and human spatial mobility across high-risk regions—and assessed in relation to ground truth data, such as rates of HIV prevalence and incidence. And, generally speaking, these assessments provided evidence for the efficaciousness of the digital epidemiological approach.

However, with a more thoughtful read, our analysis revealed several trends in the current body of work that require some scrutiny and, perhaps, revision moving forward. First, we learned that, of the four types of user-generated data employed in the studies we reviewed, data from social media, in particular Twitter, were most common. The tendency to privilege Twitter data over other social media platforms was likely related to its open Application Programming Interface (API), which made it relatively straightforward to access large portions of its content for research. However, under new leadership and branding (e.g., “X”), the once free Twitter API has been suspended and replaced with a paid tier system. Undoubtedly, this change will have an impact on the development of digital epidemiological methods, if not just for the fact that Twitter data will no longer play a central role due to heightened barriers to entry. Moreover, the type of user and use of the platform has also changed under its new leadership, with regular users tweeting less frequently and heightened prevalence of misleading and inaccurate information [53]. These changes in Twitter’s userbase and content may also make it a less viable and appropriate social media platform to use for digital epidemiology studies. What remains to be seen, however, is whether digital epidemiologists will find ways to leverage data from alternative social media platforms, for example Reddit or Instagram, or whether they will be forced to abandon their use of social media data altogether as access to social media data in general grows increasingly constrained.

We also learned that a plurality of the reviewed studies was designed to contribute to HIV surveillance. On its own, this is unsurprising given that much of what we commonly associate with the field of epidemiology involves monitoring the state of the disease at the population level. However, there are other HIV-related outcomes that merit attention and for which user-generated digital data may prove useful. For example, with the insights it can provide regarding users’ daily routines and circumstances, social media data may be helpful in identifying persons who are likely to fall out of the HIV care continuum or who may be susceptible to specific types of HIV risk, like sex drug use. As digital epidemiology advances, more effort ought to be invested in determining whether user-generated data can be applied to identify individuals who are risk for these secondary outcomes, which have to be addressed if we are to bring an end to the HIV epidemic. Arguably, this type of analysis may need to be more person-centered than the macro-level analysis of much disease surveillance, which may introduce challenges in terms of recruitment, data collection, and privacy protections that population-level analysis tries to circumvent. However, a more person-centered data analytic approach is needed if we are to address the within-group heterogeneity of members of high-risk groups, such as Black MSM, who are not monolithic in their experiences and circumstances.

Although all human-subjects research necessarily involves ethical considerations, the ethical concerns associated with digital epidemiology are particularly pronounced. Although most digital epidemiological studies draw on publicly available digital data, that these data were not intended for making health-related inferences and that users are rarely aware that information about themselves are being used in this way raises concerns about privacy, autonomy, and accountability that researchers using these methods should have to contend with more explicitly. Unfortunately, our review revealed that less than a quarter of the studies elected to go beyond a generic ethics approval statement in their papers. We contend that this must change if we are to move the field of digital epidemiology forward. To assuage public and patient concerns about digital surveillance and privacy, they must be able to see the value of this work to their own health and well-being. This means that researchers must be willing to self-reflect on their own practices and engage in these challenging conversations in their presentation of findings and directly with members of the public.

Finally, on a more practical note, most of the studies included in this review were in feasibility stages of development. As such, the collective impact of digital epidemiological methods on HIV surveillance, prevention, and treatment efforts is, thus far, limited. An important next step will be transitioning these methods from the lab into practice, for example, through demonstration projects in real-world community settings.

## Limitations

Our study had certain limitations. Although best efforts were made to include all relevant papers, it is possible that some studies were missed. For example, we chose a slightly conservative search strategy in order to exclude at the onset papers that used social media for recruitment in HIV studies, which we do not consider a form of digital epidemiology. That said, it is possible that this strategy had the unintended consequence of missing papers that did not use key terminology such as “digital epidemiology” or “infodemiology” but that would otherwise be deemed as relevant demonstrations of digital epidemiological methods. Relatedly, the search was conducted using English-language search terms, thus non-English studies were not reviewed. Finally, we focused our review on evaluating the application of user-generated digital data in HIV epidemiological studies and, therefore, refrained from going into detail about the results of the studies or in making judgements about the conclusions drawn by researchers. Future outcomes-focused investigations will be important for improving models and optimizing feature selection.

## Conclusion

Achieving goals to end the HIV epidemic worldwide demands having access to timely and accurate systems of HIV surveillance and the ability to leverage those systems to increase linkage to care in most-susceptible populations. Existing surveillance systems, however, suffer from an over-reliance on retrospective survey and interview data and, as a consequence, are prone to reporting biases and are poorly suited for facilitating timely interventions. Harnessing insights from passively observed near-real-time user-generated data, for example, social media posts, web search queries, and mobile phone call records, has been extoled as a promising way to overcome some of these limitations. Our study indicates that the anticipated epidemiological value of user-generated digital data has merit. However, this work will have limited impact if efforts are not scaled and integrated more broadly into ongoing surveillance programs. For example, public health departments could add to their existing surveillance toolkit the use of validated machine learning classifiers to detect population-level spikes in HIV-related social media posts or web search queries, which would enable more rapid public health responses in locations where these spikes occur. At the same time, however, there are growing concerns over the ethics of exploiting user-generated data for public health surveillance that need to be thoughtfully and proactively considered if broad deployment of these methods is to be beneficial, equitable, and just. As such, future research should reckon with these considerations more explicitly, and the development and implementation of digital surveillance programs ought to be informed and guided by partnerships between HIV epidemiologists, data scientists, digital ethicists, and community stakeholders.

### Supplementary Information

Below is the link to the electronic supplementary material.

Supplementary file1 (DOCX 40 KB)

## Data Availability

Not applicable.
