# Supplementary material for: Digital Epidemiological Approaches in HIV Research: a Scoping Methodological Review
Source: Curr HIV/AIDS Rep. 2023 Nov 2;20(6):470–80. doi: 10.1007/s11904-023-00673-x (PMC10719139; doi:10.1007/s11904-023-00673-x)
Supplement: Supplementary file 1 — Supplementary file1 (DOCX 40 KB) [file 11904_2023_673_MOESM1_ESM.docx]

**Appendix**

**Supplemental Table 1. Digital epidemiological studies included in the scoping review**

| **Reference** | **Author** | **Geographic Focus** | **HIV Priority Population** | **Type of digital data** |
| --- | --- | --- | --- | --- |
| [1] | Adrover et al. (2015) | None specified | None specified | Social media (Twitter) |
| [2] | Brdar et al. (2015) | Africa (Ivory Coast) | None specified | Mobile phone data |
| [3] | Burgess et al. (2022) | None specified | None specified | Social media (Twitter) |
| [4] | Cai et al. (2020) | North America (U.S.) | None specified | Social media (Twitter) |
| [5] | Chiu et al. (2017) | East Asia (China) | MSM | Web search query (Google) |
| [6] | Cuomo et al. (2020) | North America (U.S.) | PWID | Social media (Twitter) |
| [7] | Domnich et al. (2014) | Europe (Russia) | None specified | Web search query (Yandex) |
| [8] | Dong et al. (2019) | East Asia (China) | MSM | Social media (Baidu Tieba) |
| [9] | Duncan et al. (2018) | North America (U.S.) | MSM | Other GPS technology |
| [10] | Duncan et al. (2020) | North America (U.S.) | MSM | Other GPS technology |
| [11] | Godinez et al. (2023) | None specified | MSM, TGW | Social media (Reddit) |
| [12] | Ireland et al. (2015) | North America (U.S.) | None specified | Social media (Twitter) |
| [13] | Ireland et al. (2016) | North America (U.S.) | None specified | Social media (Twitter) |
| [14] | Isdory et al. (2015) | Africa (Kenya) | None specified | Mobile phone data |
| [15] | Jha et al. (2017) | North America (U.S.) | MSM | Social media (Twitter) |
| [16] | Kapur et al. (2014) | South Asia (India) | MSM | Mobile phone data |
| [17] | Li, X. et al. (2023) | East Asia (China) | None specified | Web search query (Baidu) |
| [18] | Li, Z. et al. (2021) | North America (U.S.) | None specified | Social media (Twitter) |
| [19] | Ling et al. (2016) | North America (Canada) | None specified | Web search query (Google) |
| [20] | Mavragani et al. (2018) | North America (U.S.) | None specified | Web search query (Google) |
| [21] | Nielsen et al. (2017) | South America (Brazil) | None specified | Social media (Twitter) |
| [22] | Nsabimana et al. (2018) | Africa (Rwanda) | None specified | Mobile phone data |
| [23] | Ornos et al. (2023) | None specified | None specified | Web search query (Google) |
| [24] | Park (2021) | North America (U.S.) | None specified | Web search query (Google) |
| [25] | Stevens et al. (2020) | North America (U.S.) | Youth/young adults | Social media (Twitter) |
| [26] | Thangarajan et al. (2015) | North America (U.S.) | MSM | Social media (Twitter) |
| [27] | Valdano et al. (2021) | Africa (Namibia) | None specified | Mobile phone data |
| [28] | Valdano et al. (2022) | Africa (Namibia) | None specified | Mobile phone data |
| [29] | van Heerden et al. (2020) | Africa (South Africa) | None specified | Social media (Twitter, Instagram, YouTube) |
| [30] | Weibel et al. (2017) | North America (U.S.) | MSM; TGW; PWID | Social media (Twitter) |
| [31] | Xu et al. (2022) | None specified | None specified | Social media (Twitter, Instagram, YouTube, Reddit, Tumblr) |
| [32] | Xu et al. (2023) | None specified | None specified | Social media (Twitter, Instagram, Reddit) |
| [33] | Young, LE et al. (2019) | North America (U.S.) | MSM | Social media (Facebook) |
| [34] | Young, LE et al. (2022) | North America (U.S.) | MSM | Social media (Facebook) |
| [35] | Young, SD et al. (2014) | North America (U.S.) | None specified | Social media (Twitter) |
| [36] | Young, SD et al. (2017) | North America (U.S.) | None specified | Web search query (Google) |
| [37] | Young, SD et al. (2018) | North America (U.S.) | None specified | Social media (Twitter) |
| [38] | Zhang et al. (2018) | East Asia (China) | None specified | Web search query (Baidu) |
| [39] | Zheluk et al. (2013) | Europe (Russia) | None specified | Web search query (Google, Yandex) |

**Abbreviations:** MSM – Men who have sex with men; TGW – Transgender women; PWID – Persons who inject drugs

**References**

1. Adrover C, Bodnar T, Huang Z, Telenti A, Salathé M. Identifying Adverse Effects of HIV Drug Treatment and Associated Sentiments Using Twitter. JMIR Public Health Surveill. 2015;1(2):e7. doi: 10.2196/publichealth.4488.

2. Brdar S, Gavrić K, Ćulibrk D, Crnojević V. Unveiling Spatial Epidemiology of HIV with Mobile Phone Data. Scientific Reports. 2016;6. doi: 10.1038/srep19342.

3. Burgess R, Feliciano JT, Lizbinski L, Ransome Y. Trends and Characteristics of #HIVPrevention Tweets Posted Between 2014 and 2019: Retrospective Infodemiology Study. JMIR Public Health Surveill. 2022;8(8):e35937. doi: 10.2196/35937.

4. Cai M, Shah N, Li J, Chen W-H, Cuomo RE, Obradovich N, et al. Identification and characterization of tweets related to the 2015 Indiana HIV outbreak: A retrospective infoveillance study. PLOS ONE. 2020;15(8):e0235150. doi: 10.1371/journal.pone.0235150.

5. Chiu APY, Lin Q, He D. News trends and web search query of HIV/AIDS in Hong Kong. PLOS ONE. 2017;12(9):e0185004. doi: 10.1371/journal.pone.0185004.

6. Cuomo RE, Cai M, Shah N, Li J, Chen WH, Obradovich N, et al. Characterising communities impacted by the 2015 Indiana HIV outbreak: A big data analysis of social media messages associated with HIV and substance abuse. Drug and Alcohol Review. 2020;39(7):908-13. doi: 10.1111/dar.13091.

7. Domnich A, Arbuzova EK, Signori A, Amicizia D, Panatto D, Gasparini R. Demand-based web surveillance of sexually transmitted infections in Russia. International Journal of Public Health. 2014;59(5):841-9. doi: 10.1007/s00038-014-0581-7.

8. Dong Y, Zhou X, Lin Y, Pan Q, Wang Y. HIV-related posts from a Chinese internet discussion forum: An exploratory study. PLOS ONE. 2019;14(2):e0213066. doi: 10.1371/journal.pone.0213066.

9. Duncan DT, Chaix B, Regan SD, Park SH, Draper C, Goedel WC, et al. Collecting Mobility Data with GPS Methods to Understand the HIV Environmental Riskscape Among Young Black Men Who Have Sex with Men: A Multi-city Feasibility Study in the Deep South. AIDS Behav. 2018;22(9):3057-70. doi: 10.1007/s10461-018-2163-9.

10. Duncan DT, Regan SD, Park SH, Goedel WC, Kim B, Barton SC, et al. Assessment of spatial mobility among young men who have sex with men within and across high HIV prevalence neighborhoods in New York city: The P18 neighborhood study. Spatial and Spatio-temporal Epidemiology. 2020;35:100356. doi: 10.1016/j.sste.2020.100356.

11. Godinez H, Xu Q, McMann TJ, Li J, Mackey TK. Analysis of online user discussions on Reddit associated with the transition of use between HIV PrEP therapy. Frontiers in Public Health. 2023;11:1073813.

12. Ireland ME, Schwartz HA, Chen Q, Ungar LH, Albarracín D. Future-oriented tweets predict lower county-level HIV prevalence in the United States. Health Psychol. 2015;34s:1252-60. doi: 10.1037/hea0000279.

13. Ireland ME, Chen Q, Schwartz HA, Ungar LH, Albarracin D. Action Tweets Linked to Reduced County-Level HIV Prevalence in the United States: Online Messages and Structural Determinants. AIDS and Behavior. 2016;20(6):1256-64. doi: 10.1007/s10461-015-1252-2.

14. Isdory A, Mureithi EW, Sumpter DJT. The Impact of Human Mobility on HIV Transmission in Kenya. PLOS ONE. 2015;10(11):e0142805. doi: 10.1371/journal.pone.0142805.

15. Jha D, Skums P, Zelikovsky A, Khudyakov Y, Singh R. Modeling the Spread of HIV and HCV Infections Based on Identification and Characterization of High-Risk Communities Using Social Media. Springer International Publishing; 2017. p. 425-30.

16. Kapur A, Schneider JA, Heard D, Mukherjee S, Schumm P, Oruganti G, et al. A Digital Network Approach to Infer Sex Behavior in Emerging HIV Epidemics. PLoS ONE. 2014;9(7):e101416. doi: 10.1371/journal.pone.0101416.

17. Li X, Tang K. The Effects of Online Health Information–Seeking Behavior on Sexually Transmitted Disease in China: Infodemiology Study of the Internet Search Queries. J Med Internet Res. 2023;25:e43046. doi: 10.2196/43046.

18. Li Z, Qiao S, Jiang Y, Li X. Building a social media-based HIV risk behavior index to inform the prediction of HIV new diagnosis: a feasibility study. Aids. 2021;35(Suppl 1):S91-s9. doi: 10.1097/qad.0000000000002787.

19. Ling R, Lee J. Disease Monitoring and Health Campaign Evaluation Using Google Search Activities for HIV and AIDS, Stroke, Colorectal Cancer, and Marijuana Use in Canada: A Retrospective Observational Study. JMIR Public Health and Surveillance. 2016;2(2):e156. doi: 10.2196/publichealth.6504.

20. Mavragani A, Ochoa G. Forecasting AIDS prevalence in the United States using online search traffic data. Journal of Big Data. 2018;5(1). doi: 10.1186/s40537-018-0126-7.

21. Nielsen RC, Luengo-Oroz M, Mello MB, Paz J, Pantin C, Erkkola T. Social Media Monitoring of Discrimination and HIV Testing in Brazil, 2014–2015. AIDS and Behavior. 2017;21(S1):114-20. doi: 10.1007/s10461-017-1753-2.

22. Nsabimana AP, Uzabakiriho B, Kagabo DM, Nduwayo J, Fu Q, Eng A, et al. Bringing Real-Time Geospatial Precision to HIV Surveillance Through Smartphones: Feasibility Study. JMIR Public Health and Surveillance. 2018;4(3):e11203. doi: 10.2196/11203.

23. Ornos EDB, Tantengco OAG, Abad CLR. Global Online Interest in HIV/AIDS care Services in the time of COVID-19: A Google Trends Analysis. AIDS and Behavior. 2023;27(6):1998-2004. doi: 10.1007/s10461-022-03933-w.

24. Park YJ. A socio-technological model of search information divide in US cities. Aslib Journal of Information Management. 2021;73(2):144-59.

25. Stevens R, Bonett S, Bannon J, Chittamuru D, Slaff B, Browne SK, et al. Association Between HIV-Related Tweets and HIV Incidence in the United States: Infodemiology Study. Journal of Medical Internet Research. 2020;22(6):e17196. doi: 10.2196/17196.

26. Thangarajan N, Green N, Gupta A, Little S, Weibel N. Analyzing social media to characterize local HIV at-risk populations. Proceedings of the conference on Wireless Health. Bethesda, MD: ACM; 2015.

27. Valdano E, Okano JT, Colizza V, Mitonga HK, Blower S. Using mobile phone data to reveal risk flow networks underlying the HIV epidemic in Namibia. Nature Communications. 2021;12(1). doi: 10.1038/s41467-021-23051-w.

28. Valdano E, Okano JT, Colizza V, Mitonga HK, Blower S. Using mobile phone data to identify social-sexual communities: Implications for controlling HIV epidemics. The Lancet HIV. 2022;9(12):e820-e1. doi: 10.1016/S2352-3018(22)00332-0.

29. Van Heerden A, Young S. Use of social media big data as a novel HIV surveillance tool in South Africa. PLOS ONE. 2020;15(10):e0239304. doi: 10.1371/journal.pone.0239304.

30. Weibel N, Desai P, Saul L, Gupta A, Little S. HIV risk on twitter: The ethical dimension of social media evidence-based prevention for vulnerable populations. Proceedings of the 50th Hawaii International Conference on System Sciences. 2017.

31. Xu Q, Nali MC, McMann T, Godinez H, Li J, He Y, et al. Unsupervised Machine Learning to Detect and Characterize Barriers to Pre-exposure Prophylaxis Therapy: Multiplatform Social Media Study. JMIR Infodemiology. 2022;2(1):e35446. doi: 10.2196/35446.

32. Xu Q, McMann T, Godinez H, Nali MC, Li J, Cai M, et al. Impact of COVID-19 on HIV Prevention Access: A Multi-platform Social Media Infodemiology Study. AIDS and Behavior. 2023;27(6):1886-96. doi: 10.1007/s10461-022-03922-z.

33. Young LE, Fujimoto K, Schneider JA. Facebook group affiliation ties, group topics, and HIV behavioral characteristics among young Black men who have sex with men: Potential for public health intervention. SSM-Population Health. 2019;9. doi: 10.1016/j.ssmph.2019.100510.

34. Young LE, Tang JL, Nan Y. Social Media Communication and Network Correlates of HIV Infection and Transmission Risks Among Black Sexual Minority Men: Cross-sectional Digital Epidemiology Study. JMIR Formative Research. 2022;6(10):e37982. doi: 10.2196/37982.

35. Young SD, Rivers C, Lewis B. Methods of using real-time social media technologies for detection and remote monitoring of HIV outcomes. Preventive medicine. 2014;63:112-5. doi: 10.1016/j.ypmed.2014.01.024.

36. Young SD, Yu W, Wang W. Toward automating HIV identification: Machine learning for rapid identification of HIV-related social media data. Journal of Acquired Immune Deficiency Syndromes. 2017;74:S128-S31. doi: 10.1097/QAI.0000000000001240.

37. Young SD, Zhang Q. Using search engine big data for predicting new HIV diagnoses. PLOS ONE. 2018;13(7):e0199527. doi: 10.1371/journal.pone.0199527.

38. Zhang Q, Chai Y, Li X, Young SD, Zhou J. Using internet search data to predict new HIV diagnoses in China: a modelling study. BMJ Open. 2018;8(10):e018335. doi: 10.1136/bmjopen-2017-018335.

39. Zheluk A, Quinn C, Hercz D, Gillespie JA. Internet Search Patterns of Human Immunodeficiency Virus and the Digital Divide in the Russian Federation: Infoveillance Study. J Med Internet Res. 2013;15(11):e256. doi: 10.2196/jmir.2936.
